# Characterization of Small HSPs from *Anemonia viridis* Reveals Insights into Molecular Evolution of Alpha Crystallin Genes among Cnidarians

**DOI:** 10.1371/journal.pone.0105908

**Published:** 2014-09-24

**Authors:** Aldo Nicosia, Teresa Maggio, Salvatore Mazzola, Fabrizio Gianguzza, Angela Cuttitta, Salvatore Costa

**Affiliations:** 1 Laboratory of Molecular Ecology and Biotechnology, National Research Council-Institute for Marine and Coastal Environment (IAMC-CNR) Detached Unit of Capo Granitola, Torretta Granitola, Trapani, Italy; 2 Institute for Environmental Protection and Research-ISPRA, Palermo, Italy; 3 National Research Council-Institute for Marine and Coastal Environment (IAMC-CNR), Calata Porta di Massa, Napoli, Italy; 4 Dipartimento Scienze e Tecnologie Biologiche Chimiche e Farmaceutiche, University of Palermo, Palermo, Italy; Stazione Zoologica Anton Dohrn, Naples, Italy

## Abstract

Gene family encoding small Heat-Shock Proteins (sHSPs containing α-crystallin domain) are found both in prokaryotic and eukaryotic organisms; however, there is limited knowledge of their evolution. In this study, two small *HSP* genes termed *AvHSP28.6* and *AvHSP27*, both organized in one intron and two exons, were characterised in the Mediterranean snakelocks anemone *Anemonia viridis*. The release of the genome sequence of *Hydra magnipapillata* and *Nematostella vectensis* enabled a comprehensive study of the molecular evolution of α-crystallin gene family among cnidarians. Most of the *H. magnipapillata sHSP* genes share the same gene organization described for *AvHSP28.6* and *AvHSP27,* differing from the s*HSP* genes of *N. vectensis* which mainly show an intronless architecture. The different genomic organization of *sHSPs*, the phylogenetic analyses based on protein sequences, and the relationships among Cnidarians, suggest that the *A.viridis sHSPs* represent the common ancestor from which *H. magnipapillata* genes directly evolved through segmental genome duplication. Additionally retroposition events may be considered responsible for the divergence of *sHSP* genes of *N. vectensis* from *A. viridis.* Analyses of transcriptional expression profile showed that *AvHSP28.6* was constitutively expressed among different tissues from both ectodermal and endodermal layers of the adult sea anemones, under normal physiological conditions and also under different stress condition. Specifically, we profiled the transcriptional activation of *AvHSP28.6* after challenges with different abiotic/biotic stresses showing induction by extreme temperatures, heavy metals exposure and immune stimulation. Conversely, no *AvHSP27* transcript was detected in such dissected tissues, in adult whole body cDNA library or under stress conditions. Hence, the involvement of *AvHSP28.6* gene in the sea anemone defensome is strongly suggested.

## Introduction

The small heat-shock proteins (sHSPs) represent one of the most widespread but also poorly conserved family of molecular chaperones [1]–[3]. As first reported by Ingolia and Craig [4], these low-molecular-weight proteins are evolutionarily related to the vertebrate eye lens protein because of the presence of a conserved α-crystallin domain consisting of 80–100 amino acid residues and are defined on the basis of its conserved β-sandwich structure composed of antiparallel β-sheets [5]. The sHSPs are constitutive and stress-inducible proteins with variable masses between 12 and 43 kDa and usually form large aggregates under normal physiological conditions. They are ubiquitously expressed to perform a plethora of housekeeping functions; in response to stress, the oligomeric structure dissociates into suboligomeric species [6], [7] and hydrophobic sites are exposed to suppress protein aggregation, promoting the restoration of cellular homeostasis [5], [8], [9].

In addition to their central role as chaperones, sHSPs are also involved in a number of cellular activities, including cell proliferation and differentiation, cytoskeletal organisation and integrity, apoptosis and immune response [10]–[12]. Indeed sHSPs have been shown to regulate redox state enzymes activity and to modulate cell death pathways by activation of caspase-3, inactivation of Bax and blocking the release of Smac and cytochrome C [10]. Moreover, it has been shown that the functions of sHSPs are highly effective against pathological conditions such as cardiac hypertrophy and Alzheimer's disease [13].

Through the years, several studies investigating sHSPs in vertebrates and invertebrates, including mammals, rodents, aves, amphibians and nematodes, have been reported. Recently, sHSPs have been cloned from molluscs and insects and their involvement in mechanisms of stress response have been characterised.

In abalone *Haliotis discus discus* mRNA level of *HdHSP20* was up-regulate after exposure to thermal shock, extreme salinities, heavy metals and the microbial infections [14]. Similarly, in scallop *Argopecten irradians*, *AiHSP22* mRNA showed over expression in response to heavy metal exposure [15]. In *Drosophila melanogaster* the mRNA levels of several sHSPs including, *HSP22*, *HSP23, HSP26*, and *HSP27*, were induced by cold exposure [16] and in *Apis cerana cerana* the expression of *AccHSP27.6* transcript was induced by exposure to heat shock, chemicals and microbial challenge [17]. Moreover, in common cutworm *Spodoptera litura HSP20.4* and *HSP20.8* genes were upregulated during midgut metamorphosis[18].

However, there is little characterisation of sHSPs in cnidarians [19], [20], and to date, no sHSPs have been studied in Mediterranean snakelocks sea anemone.

The sea anemone *Anemonia viridis* (Anthozoa: Actinaria) is a widespread and extensively studied Mediterranean species of Cnidaria [21]–[23].

This phylum is sister taxa of Bilateria within the Eumetazoan and some studies demonstrated that time of divergence between Cnidarians and Bilaterians dates back to 600 million years ago in the Precambrian era [24]–[26]. The phylum split into two major lineages: the class Anthozoa to which *Anemonia viridis* belongs and its sister group, the Medusozoa [27], [28].

Within the Cnidaria, the Class Anthozoa is phylogenetically basal, and both morphological and molecular evidence are consistent with the idea that members of this class best represent the primitive cnidarians and are therefore least changed from the Ureumetazoan.

The Medusozoa comprises three classes Scyphozoa (true jellyfishes), Cubozoa (box jellyfishes) and Hydrozoa (hydras and hydromedusae).

Cnidaria includes some of the most morphologically simple organisms such as sea anemones and corals; they are primarily characterized by a single body axis, only two germ layers (diploblastic organism) and two-to-three cell lineages. All these characteristics led to the assumption of underlying genetic simplicity, but recent genomic and transcriptional surveys on cnidarians have revealed surprising ancestral complexity in the genomes of these simple animals [29]–[30].

In particular, many genes that were previously thought to have originated within vertebrates because of their absence in the invertebrates genomes have been found in cnidarians. These genes must have been present in the cnidarian-bilaterian ancestor, some 634 million years ago. [31]–[32]

Recently, the development of a large dataset of *A*. *viridis* ESTs (>39000 RNA sequences) has attracted much interest supporting *in vitro* omic experiments [33]–[36]. In addition, the efficient separation of the ectoderm from the endodermal layers of sea anemones represents a tool for profiling the tissue-specific expression of selected genes.

In the present work, an open reading frame (ORF) for a sHSP, herein designated as *AvHSP28.6*, was recovered from the *A*. *viridis* EST collection, and its genomic organisation was characterised.

In addition to the *AvHSP28.6*, a second unexpressed gene that may encode a sHSP, herein described as *AvHSP27*, was isolated from genomic DNA. The ancient phylum divergence and the availability of whole genome sequences of two basal cnidarians, *Hydra magnipapillata* [37] and *Nematostella vectensis* [29], allowed us to analyse the molecular evolution of the α-crystallin gene family among cnidarians. Moreover, qRT-PCR assays indicate that the transcriptional expression of *AvHSP28.6* responds to a number of environmental stressors (including extreme temperature, heavy metals and immune system stimulation), suggesting its involvement in the sea anemone defensome [19].

## Materials and Methods

### Ethics Statement

*A. viridis* housing and husbandry were performed in accordance with the best practices dveloped in the cnidarian community to optimise animal health. No specific permissions were required for the locations/activities because *A. viridis* is not classified an endangered or protected species. Housing and husbandry of *A. viridis* were carried out in accordance to the best practices developed in the cnidarians community in order to optimize animal health. However, all facilities and procedures complied with the Directive 2010/63/EU and the ARRIVE guidelines.

### GenBank Accession Numbers

The s*HSP* sequences were obtained from the *A*. *viridis* EST database [38] available at the National Centre for Biotechnology Information (NCBI) under the following accession numbers: FK753571, FK734577 and FK735326. The *sHSP* gene sequences of *N. vectensis* were extracted from the nucleotide dataset under the following gene IDs: 5519939, 5500827, 5519551, 550032, 5519484 and 5504814. Similarly, the *sHSP* gene sequences of *H. magnipapillata* were obtained from the nucleotide dataset under the following gene IDs: 100202946, 100200369, 100214931, 100204018, 100205528, 100199462, 100197458, 100211336, 100199246, 101235243 and 100211409. Protein IDs for sHSPs from *N. vectensis* and *H. magnipapillata* are reported in Table 1.

**Table 1:** sHSPs from cnidarians used for phylogenetic analysis with their GenBank Accession Number and References.

Species	Gene Reference	GeneBank Accession Number	References
*N. vectensis*	Alpha-crystallin domain (ACD)	XP_001639719	[29]
	Alpha-crystallin domain (ACD)	XP_001622212	
	Alpha-crystallin domain (ACD)	XP_001639451	
	Alpha-crystallin-Hsps_p23-like	XP_001621785	
	Alpha-crystallin domain (ACD)	XP_001639372	
	Alpha-crystallin domain (ACD)	XP_001625685	
*H. magnipapillata*	Heat shock protein Hsp-16.2-like	XP_002166274	[37]
	Heat shock protein Hsp-16.1-like	XP_004212222	
	Heat shock protein Hsp-16.2-like	XP_002160804	
	Alpha-crystallin domain (ACD)	XP_004207394	
	Alpha-crystallin domain (ACD)	XP_002160685	
	Alpha-crystallin domain (ACD)	XP_002160720	
	Alpha-crystallin domain (ACD)	XP_002157141	
	Alpha-crystallin A chain-like	XP_002166332	
	Alpha-crystallin B chain-like	XP_002166333	
	Alpha-crystallin domain (ACD)	XP_002166334	
	Alpha-crystallin A chain-like	XP_002166335	

### Nucleic Acids Extraction and First-Strand cDNA Synthesis

Specimens of A. viridis were collected from the Capo Granitola Coast (Torretta Granitola, Latitude 37°34'30.00"N Longitude 012°40'47.26"E, Trapani, Italy) in the south of Sicily and maintained in Millipore Filtered Sea Water (MFSW) at 18±1°C with a 12·h:12·h light:dark photoperiod. Water quality parameters (pH, salinity, nitrate, and ammonium levels) were monitored with kits to measure the water quality of the aquarium.

*A. viridis* samples were flash frozen in liquid nitrogen and ground to fine powder using a tissue disruptor. For DNA extraction, the powder was dissolved in a DNAzol reagent (Invitrogen Corporation, Carlsbad, CA, USA), and further genomic DNA purification steps were performed according to the manufacturer's instructions. DNA concentrations and quality were verified by spectrophotometry (optical density (OD) at 260 nm), whereas the integrity was checked using a 0.8% agarose gel. The DNA was stored at −20°C for future use.

For RNA purification, tentacles and body walls were cut lengthwise and scraped to separate the endodermal cell layer and the film of mesogloea from the ectodermal cells. The small size of the pharynx, muscles and gonads prevented the separation of ectodermal from endodermal tissue; consequently, RNA was isolated from both ectodermal and endodermal layers. Tissues were frozen in liquid nitrogen and ground into a fine powder using a tissue disruptor. The powder was dissolved in Trizol reagent (Invitrogen Corporation, Carlsbad, CA, USA), and further RNA purification steps were performed according to the manufacturer's instructions. RNA concentrations and quality were verified by spectrophotometry (OD at 260 nm), whereas the RNA integrity was checked using a 1.5% agarose gel. The RNA was stored at −80°C for future use. The extracted RNA (2 µg) was treated with RNA qualified 1 (RQ1) RNase-Free DNase (Promega, Madison, WI, USA) to remove any residual genomic DNA contamination, and the DNase was inactivated by adding 25 mM EDTA. First-strand cDNA was synthesised from 2 µg DNase-treated total RNA samples using oligo(dT)_18_ and Superscript III (Invitrogen Corporation, Carlsbad, CA, USA) following the manufacturer's instructions. The cDNA mixture was stored at −20°C. RNA cross-contamination was assessed by performing Reverse Transcriptase-Polymerase Chain Reactions (RT-PCRs) using CA2-M and HMG-R specific primers (Table 2), and the expected bands were obtained. The cDNA derived from the ectodermal tissues, endodermal tissues or whole body were separately subjected to PCRs using a pair of primers (qHSP-F and qHSP28.6-R or qHSP27) specifically chosen to ensure the amplification of the two sequence variants. PCRs were performed using Platinum Taq DNA Polymerase (Invitrogen Corporation, Carlsbad, CA, USA) with the following conditions: pre-denaturation at 95°C for 1 min and 25 cycles of 94°C for 30 s, 60°C for 30 s, and 72°C for 30 s, followed by elongation at 72°C for 2 min.

**Table 2:** Oligonucleotide primers used in this study.

Primers	Sequences (5′–3′)	Amplicon size (bp)
HSP28.6-F	CGTGGGCCTTGTTTCGTATC	686
HSP28.6-R	GGAGTTTCGGGTTTTCATCG	
3′Race	CGATGAAAACCCGAAACTCC	704
sHSPg-F	GTTAAGGCAAAGCACTAACGTG	1808 (AvHSP28.6)
sHSPg-R	GTATTGGTATGTGGGAGGTG	1544 (AvHSP27)
18S-F	AAGCTCGTAGTTGGATTTCGG	161
18S-R	CTGGCCTGCTTTGAACACTC	
RCC2-F	GGTTCCAAATCCTCCACAAACC	83
RCC2-R	TGTCCCAATCCGCACGTTAC	
COP γ-F	GCCTGTTGGACACCGATGAT	142
COP γ-R	TGCAAGGCTCTCTCCAGTCC	
qHSP-F	GTCAAAATGAGCACTGTTAAGGC	193
qHSP28.6-R*	TGTCCGGATTCATGGAGTTTC	
HSP27-R*	CGTCCAAGTGCCTTAGCTTGC	158
CA2-m-F	CTTTGGCGGCATTTCACTTG	129
CA2-m-R	GTGATTGGTTGGAGCCATCG	
HMG-F	AGTATGTGAAGCCATAGTGC	311
HMG-R	TAGTACCACCACCAACAGTC	

*qHSP-F and qHSP28.6-R were used for the selective amplification of HSP28.6;

qHSP-F and qHSP27-R were used for the selective amplification of HSP27.

### Cloning of AvHSP28.6 and AvHSP27 Genes

A pair of primers, HSP28.6-F and HSP28.6- R (Table 2), was used to clone the partial cDNA sequence of AvHSP28.6. A PCR amplification was performed using Platinum Taq DNA Polymerase (Invitrogen Corporation, Carlsbad, CA, USA) under the following conditions: pre-incubation at 95°C for 2 min; 35 cycles consisting of denaturation at 95°C for 30 s, annealing at 50°C for 30 s and extension at 72°C for 30 s; and a final extension at 72°C for 2 min. The amplified product was analysed on a 1% agarose gel and subcloned into the pGEM-T Easy vector (Promega, USA), and the nucleotide sequence were verified using T7 and SP6 primers.

Based on the partial sequence of *AvHSP28.6* cDNA, the 3' end was obtained by PCR-RACE using the SMART RACE cDNA application kit (Clontech, USA) as described in the user manual. The product was cloned into the pGEM-T Easy vector (Promega, USA) and transformed into DH10B *E. Coli* cells (Promega, USA). Plasmid DNA, from three independent clones, was recovered using the QIAprep Spin Miniprep Kit (QIAGEN, Japan) and sequenced using T7 and SP6 primers.

Based on the 5′- and 3′-UTR sequences, an appropriate pair of primers (sHSPg-F and sHSPg-R) was used to isolate the genomic DNA sequences.

### Sequence and Phylogenetic Analyses

Signal peptides, functional sites and domains in the predicted amino acid sequences were predicted using the Simple Modular Architecture Research Tool (SMART) program, the InterPro database, the Pfam database, the PROSITE program, and the Eukaryotic Linear Motif resource (ELM) for Functional Sites in Proteins.

An alignment of *A. viridis sHSP* nucleotide and deduced protein sequences was generated using ClustalX [39] and was manually edited. All introduced gaps were treated as missing data in subsequent analyses. The nucleotide and amino acid sequences were analysed for polymorphisms and substitution rates using DNAsp software (http://www.ub.edu/dnasp/) [40] and Molecular Evolution Genetics Analyses (MEGA) software version 6 (http://www.megasoftware.net/mega.php) [41].

To reconstruct the evolutionary diversification and the molecular evolution of the multifunctional gene family of sHSPs, we explored the variety of sHSPs in the GenBank protein database for other cnidarians and for different Eumetazoan groups (e.g. Nematodes, Crustaceans, Teleosts, etc.), deriving a phylogenetic tree representing their relationships.

Basic Local Alignment Search Tool (TBLASTN, BLASTP) analyses were performed to recover sHSPs from GenBank on the basis of the presence of the α-crystallin domain.

Phylogenetic and molecular evolutionary analyses were conducted on the amino acid sequences of the α-crystallin domain (ACD) using a Neighbour Joining (NJ) method, implemented in MEGA version 6.0, and the Maximum Parsimony (MP) method with the PROTPARS and BOOT programs of the Phylogenetic Inference Package (PHYLIP 3.4) [42]. Internal branch support was assessed using 1000 bootstrap replications. The phylogenetic trees were determined using the sHSPs from cnidarians and eumetazoans reported in Table 2 (Cnidarians) and 3 (Eumetazoans). The sHSPs from *Salpingoeca rosetta* (Choanoflagellata), *Amphimedon queenslandica* (Porifera) and *Bacillus subtilis* (Bacteria) were used as an outgroup.

**Table 3:** sHSPs from Eumetazoans, Porifera, Choanoflagellata and Bacteria used for phylogenetic Analysis with their GenBank Accession Number and References.

Species	GeneBank Accession Number	Taxonomic group	References
*Artemia franciscana*	gi83701157	Crustacea	[75]
*Aedes aegypti*	XP_001663494	Insecta	[76]
*Megachile rotundata*	XP_003701702	Insecta	Automated annotation
*Ceratitis capitata*	XP_004523813	Insecta	Automated annotation
*Haemonchus contortus*	CDJ94402	Nematoda	Laing et al. (2014) unpublished
*Caenorabditis brenneri*	EGT36913	Nematoda	Wilson et al. (2011) unpublished
*Branchiostoma floridae*	XP_002596852.	Cephalocordata	[77]
*Xiphophorus maculatus*	XP_005804010	Cephalocordata	Automated annotation
*Danio rerio*	NP_001006040	Osteichtyes	[78]
*Latimeria chalumnae*	XP_005996349	Osteichtyes	Automated annotation
*Xenopus laevis*	NP_001086558.	Amphibia	[79]
*Columba livia*	XP_005508027.	Aves	Automated annotation
*Melopsittacus undulatus*	XP_005145471	Aves	Automated annotation
*Amphimedon queenslandica*	XP_003383187	Porifera	Automated annotation
*Salpingoeca rosetta*	XP_004994845	Choanoflagellata	Russ et al. (2013) unpublished
*Bacillus subtilis*	NP_049441	Bacteria	[80]

### Challenging Sea Anemone with Different Environmental Stresses

To test the effect of heavy metals on *AvHSP28.6* and *AvHSP27* expression, sea anemones (n = 3 animals per treatment) were subjected to 12-h waterborne exposure to CdCl_2_ and PbCl_2_ at 18±1°C with a 12·h:12·h light:dark photoperiod. CdCl_2_ and PbCl_2_ solutions were prepared using 99% pure chloride salts (Sigma-Aldrich). Stock solution were prepared in Milli-Q water and diluted in MFSW to the required concentrations (2, 10, and 50 µg/L).

To test the effect of hypothermic and hyperthermic stress conditions on *AvHSP28.6* expression, sea anemones (n = 3 animals per treatment) were maintained in MFSW with a 12·h:12·h light:dark photoperiod, performing 4°C or 28°C treatments for variable lengths of time (from 0.5 h to 6 h).

To explore the influence of PAMP-induced immune response on *AvHSP28.6* expression, sea anemones (n = 3 animals per treatment) were maintained for 18 h in MFSW supplemented with 1 µg/ml lipopolysaccharide (LPS) from *E. coli* 0111:B4 (Santa Cruz Biotechnology) at a 12·h:12·h light:dark photoperiod.

### Relative Quantification Using Real-time Quantitative Polymerase Chain Reaction (RT-qPCR)

RT-qPCR was performed using the ABIPRISM 7500 System (Applied Biosystems, Forster City, USA) with Power Sybr Green as detection chemistry (Applied Biosystems, Forster City, USA). The 18S rRNA, RCC2 (Regulator of Chromosome Condensation protein 2) and COPγ (Coatomer subunit gamma) validated by Ganot et al. [33], were chosen as putative reference genes. Their expression stability among the different conditions was evaluated using the GeNorm software [43] and they were selected as internal controls in our experiments. Serial dilutions of pooled cDNAs from both control and treated samples were prepared to determine the PCR efficiency of the target and reference genes (data not shown) and amplification efficiency ranged from 1.8 to 2.1. A GeNorm normalization factor was calculated based on the expression level of these reference genes and used to quantify the expression levels of the target gene. Quantitative real-time PCR was conducted according to the manufacturer's recommended procedures, and every reaction was repeated in triplicate. The amplification conditions were the following: initial denaturation at 95°C for 10 min and 40 cycles of 95°C for 30 s and 60°C for 50 s, followed by a melting curve from 60 to 95°C. Amplicons were detected by agarose gel analysis after each PCR to confirm the amplification of the specific gene. All data represented relative mRNA expressed as the mean ± S.D. (n = 3). Significant differences between values of different treated groups and the reference control groups were determined by one-way ANOVA with Tukey's post-test.

## Results and Discussions

### *A. viridis* sHSP cDNA Characterisation

Starting from a sequence (FK734577.1) encoding a putative small HSP found in the EST database of the sea anemone *A. viridis*, a specific primer was designed and used to isolate the 3′-end of the cDNA. The putative full-length cDNA was obtained by assembling the 3′RACE product with the original EST.

**Figure 1:**
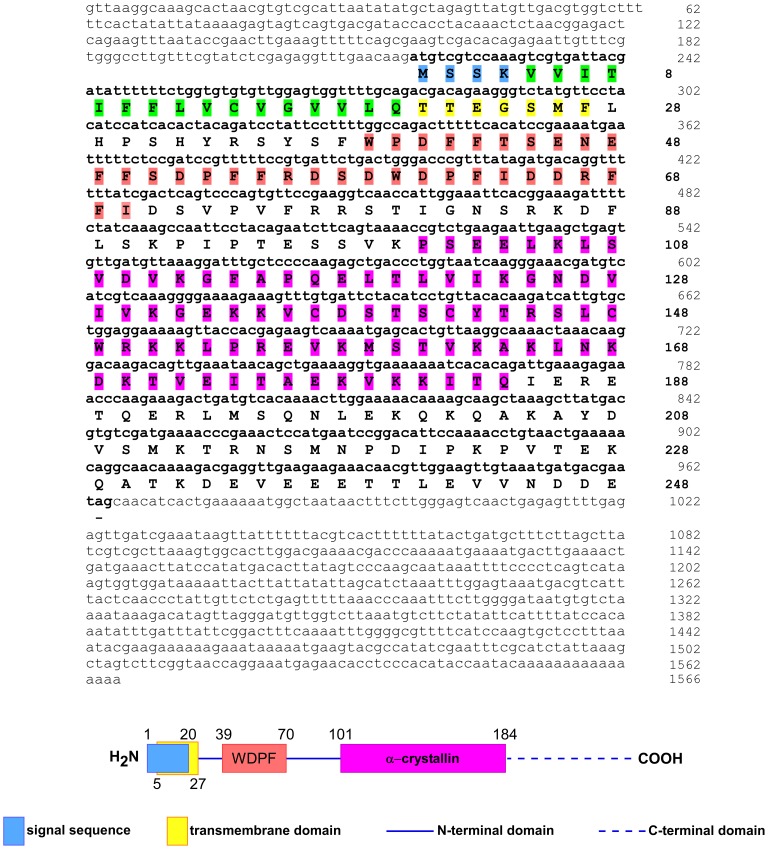
Full-length cDNA and protein sequences of sea anemone *AvHSP28.6*. The nucleotide and deduced amino acid sequence of the ORF, 5′- and 3′-UTRs were numbered on the right. The amino acids that constitute the alpha-crystallin domain (ACD) were shaded in violet. A schematic representation of HSP28.6 indicating the predicted domains is also provided.

The full-length cDNA was 1,566 bp with a 5′-untranslated region (UTR) of 218 bp and a 3′-UTR of 585 bp. Three in-frame stop codons (TAG, TAA and TGA) and a Kozak consensus (AAG) are contained in the 5′-UTR upstream of the ATG start codon, whereas a stop codon (TAG), a classical polyadenylation signal sequence (AATAAA) and a poly(A) tail were detected in the 3′-UTR (Figure 1). The cDNA contains an open reading frame (ORF) of 747 bp corresponding to 248 amino acid residues. The predicted protein has an estimated molecular mass of 28606.6 Da, a theoretical pI of 6.25, and contains an α-crystallin domain (ACD; amino acid residues 101–184) in its C-terminal region, which represents a hallmark of the sHSP family.

An analysis of the predicted secondary structure showed both an α helix content of 29.84% and stranded β-sheets consistent with previous investigations of sHSPs [3], [44].

In addition, in the consensus sequence of most sHSPs, only four cysteines are present in the protein sequence [45]; the deduced protein contains 30 glutamine and glutamic acid (Glx) residues, constituting 12.1% of the total amino acids. Such residues could provide additional electrostatic forces conferring stability even at high temperatures [46].

All these features clearly represent hallmarks for α-crystallin proteins; thus, these findings enabled us to consider this cDNA to be representative of a likely member of the sHSP family, and we designated it as *AvHSP28.6*.

Moreover, *in silico* analyses predicted the presence of an N-terminal signal sequence (amino acid residues 1–20) partially overlapped with a transmembrane domain (amino acid residues 5–27) most likely targeting the protein into the membrane or to the secretory pathway through the involvement of the endoplasmic reticulum and Golgi apparatus (Figure 1). Notably, none of the identified small HSPs contain a signal peptide or transmembrane domain. However, predictions of amphiphilic α-helices with a high hydrophobic moment in the N-termini of some small HSPs suggest that such structures may serve for insertion into membranes [47].

Indeed, members of the mammalian sHSP family were found to be associated with membranes, and several studies also showed that sHSPs may play a pivotal role in membrane quality control, contributing to the maintenance of integrity, particularly under stress conditions [48]. Thus, the signatures found in this study represent, to date, the first example of a sHSP that may be able to directly insert or interact with the biological bilayers.

Mammalian HSP27 is a component of the p38 mitogen-activated protein kinase (p38MAPK) signalling pathway, and small HSPs are involved in cell death pathways through interactions with key factors or modification of the phosphorylation status [49]. A computational search identified the putative decapeptide docking sites K^165^LNKDKTVEI^174^ for p38 MAPK and JNK in the AvHSP28.6 and AvHSP27 protein sequence. Moreover, several phosphorylation recognition sites and docking motifs for the Ser/Thr protein phosphatase identified in the AvHSP28.6 and AvHSP27 may be responsible for the regulation of both proteins.

Finally, a small prolinephenylalanine-rich region containing putative WDPF motifs, located between residues 39 and 70, was identified in both proteins (Figure 1). This motif was found in many of the sHSPs [47], [50], and it is involved in phosphorylation and oligomerisation, thus modulating the activity of ACD proteins.

Although the effective functionality of the above predicted motifs is far from being fully described, the analysis suggests that the fine mechanisms of sHSP regulation described in mammals, including post-translational modifications and protein–protein interactions, might also occur in *A. viridis*.

### Genomic Organisation and Inferences about the Molecular Evolution of sHSPs

To study the gene organisation, the genomic sequence of *AvHSP28.6* was isolated using primers corresponding to the 5′- and 3′-UTRs of the cDNA.

**Figure 2:**
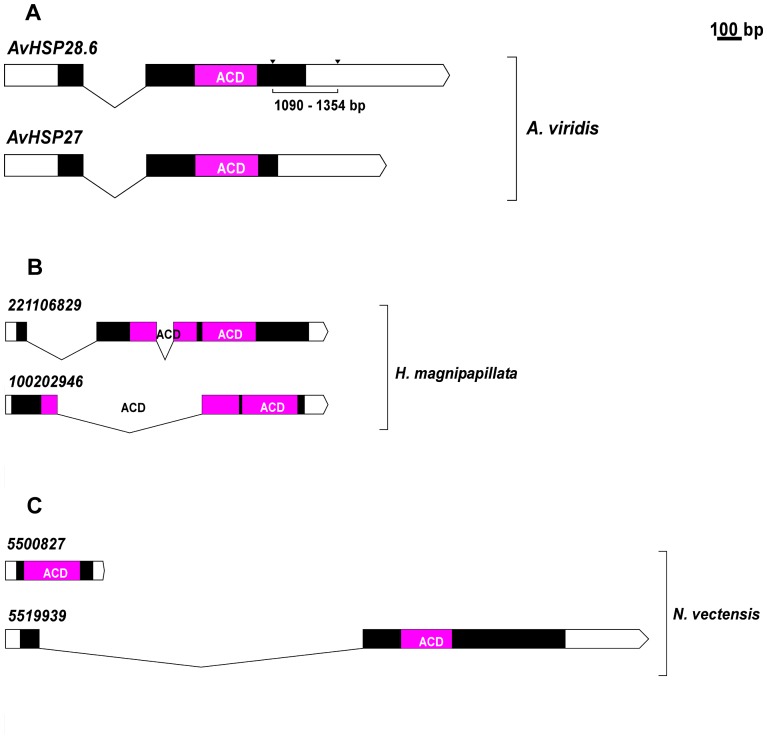
Gene structure for cnidarians *sHSPs*. The white boxes represent 3′- and 5′-untranslated regions of the mRNA, and protein coding exons are shown in black. The sequences encoding the ACD are shown in violet. **A:** exon-intron structures of *AvHSP28.6* and *AvHSP27*, the translation start sites are within exon 1 and the alpha-crystallin domain is coded within exon 2. Downward arrows specify the 264 bp deletion in *AvHSP27*. **B:** Representative exon-intron structure of two *sHSPs* from *N. vectensis*. **C:** Representative exon-intron structure of two *sHSPs* from *H. magnipapillata*.

Unexpectedly, 2 different fragments, 1.8 and 1.5 kb in length, were amplified from a single individual. A comparison of the two full-length genomic amplicons and cDNA sequences showed that each gene contained an intron (258 bp) and two exons (Figure 2A). In addition, canonical GT/AG splice junctions were found in the sequences.

**Figure 3:**
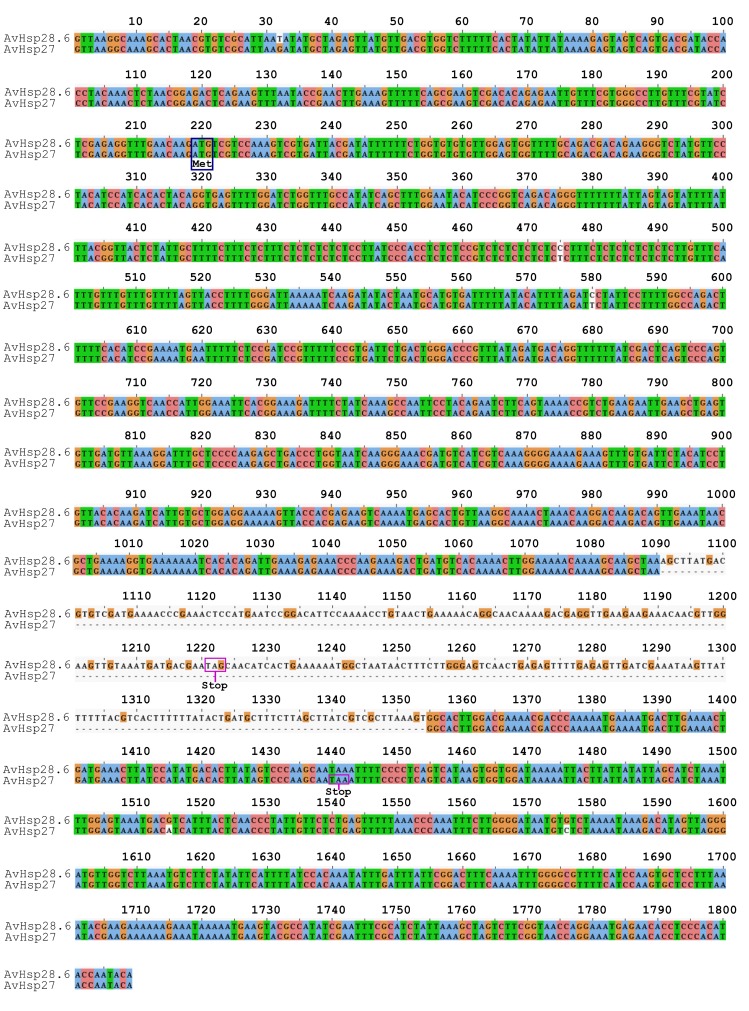
Multiple sequence alignment of *AvHSP28.6* and *AvHSP27* from *A. viridis*. The nucleotide sequences were aligned using ClustalW2. Dashes indicate gaps introduced to maximise the alignment. The ATG start codon is indicated by a blue box, while in-frame stop codons (TAG or TAA) are boxed in purple. The numbering of nucleotides refers to the alignment.

The two genes differ from one another by 5 nucleotide substitutions, 2 of which are located in the coding region (position 475 and 580 in the alignment). Furthermore, the 1.5 kb fragment (designated as *AvHSP27*) contains a deletion of 264 bp downstream of the intron acceptor site between positions 1091 and 1354 in the alignment (Figure 3). This deletion alters the reading frame, abrogates the first stop codon (TAG) and causes the continuation of protein synthesis until the downstream stop codon (TAA) is encountered. The AvHSP28.6 and AvHSP27 protein sequence alignment showed 248 amino acids, 23 of which were variable, and the consequential pairwise difference using the Poisson model resulted in 0.104.

The first exon of each gene (319 bp) corresponds to the 5′-UTR, the translation initiation codon ATG and 32 amino acids of the N- terminus, whereas the second exon (1231 bp in AvHSP28.6 and 967 bp in AvHSP27) corresponds to the ACD domain, the amino acid residues of the C-terminus and the 3′-UTR.

This gene organisation is different than that of the genomic structure of *sHSPs* orthologous from other species. In plants, yeast, and most invertebrates, the *sHSP* genes are encoded by intronless genes. In *Halocynthia*, three introns interrupt the coding sequence [1], whereas the structure usually described in mammalian (human and rodent), amphibian (*Xenopus tropicalis*), fish (*Danio rerio*), arthropods (*Ixodes scapularis*) or molluscs (*Haliotis discus discus*) includes the presence of 3 exons separated by two introns [14].

The availability of whole genome sequences of two basal cnidarians allowed us to identify the genomic sequences encoding orthologs of *AvHSP28.6* in the hydrozoan *Hydra magnipapillata* and in the anthozoan *Nematostella vectensis*. An exhaustive search for *sHSP* genes in the above mentioned cnidarians retrieved 6 and 11 orthologous in *N. vectensis* and *H. magnipapillata*, respectively. Several other predicted proteins were detected in the database but were not subjected to further analysis as they were incomplete or the sequences were considered likely to be an artefact because of the absence of any supporting ESTs.

Although our search does not map all *sHSPs* in *A. viridis*, it suggests that the Mediterranean sea anemone may have fewer members of sHSPs than *N. vectensis* and *H. magnipapillata*.

To validate this hypothesis, the relative abundances of transcripts encoding ACD in the *A. viridis* database were compared to the number of ESTs encoding the previously calculated sHSPs in others Cnidarians. Only three somewhat overlapping cDNAs encoding ACD were found amongst over 39000 ESTs for *A. viridis*.

Analyses of the gene structure allowed us to infer some evolutionary implications.

Most *H. magnipapillata sHSP* genes contain two repeats for the ACD and only one intron in close proximity or interrupting the ACD coding sequences (Figure 2B). This resembles the gene organisation described for *AvHSP28.6* and *AvHSP27*. Notably, such architecture is not the common genomic structure of *sHSPs* in *N. vectensis*. A relatively high number showed an intronless organisation, and only one gene contains an intron with two exons (Figure 2C). Because intron absence represents a hallmark of gene duplication by retroposition events, the pattern of intron gain and loss is considered a powerful tool to investigate the mechanisms of gene duplication 51]. Thus, it could be hypothesised that the *sHSPs* in *N. vectensis* originated by retrotranscription of processed mRNAs and subsequent insertion into the genome. Moreover, such analysis allowed us to putatively deduce that the origin of the diversity of *sHSP* genes in *A. viridis* and *H. magnipapillata* could arise from mechanisms of segmental genome duplication.

### Phylogenetic Analysis among Cnidarians and with Other Eumetazoa

To gain further insights and establish homology relationships, sequence similarity analyses were also performed at the protein level.

**Figure 4:**
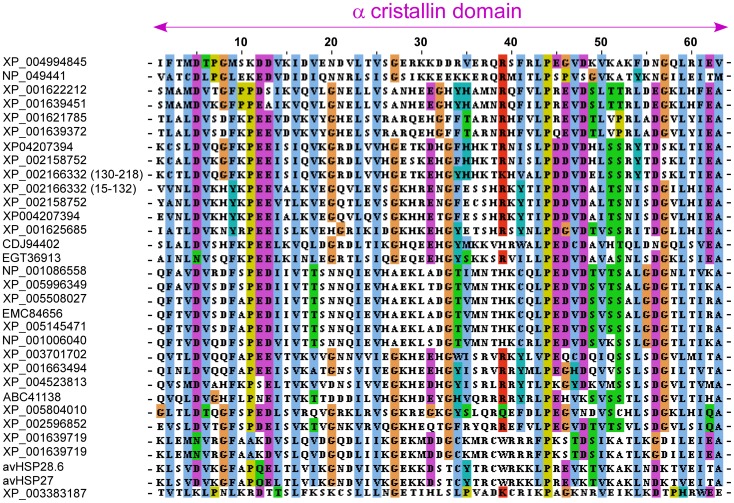
Multiple protein sequence alignment of *A. viridis* HSP28.6 and HSP27 with other cnidarians sHSPs. Only the ACD from different cnidarians sHSPs were used. Accession numbers and species are listed in Table 2.

Because the variability of the N- and C- terminal ends, a multiple sequence alignment was constructed for the ACD of *A. viridis* sHSPs and other small proteins from *N. vectensis and H. magnipapillata* (Figure 4). Such alignment resulted in 86 residues, 84 of which were variable and 78 informative. Considering the alignment of sHSPs from *A. viridis* and *N. vectensis*, 82 residues were variable, 66 of which were informative. Similarly the alignment of sHSPs from *A. viridis* and *H. magnipapillata* retrieved 79 variable amino-acid residues and 72 informative residues.

**Figure 5:**
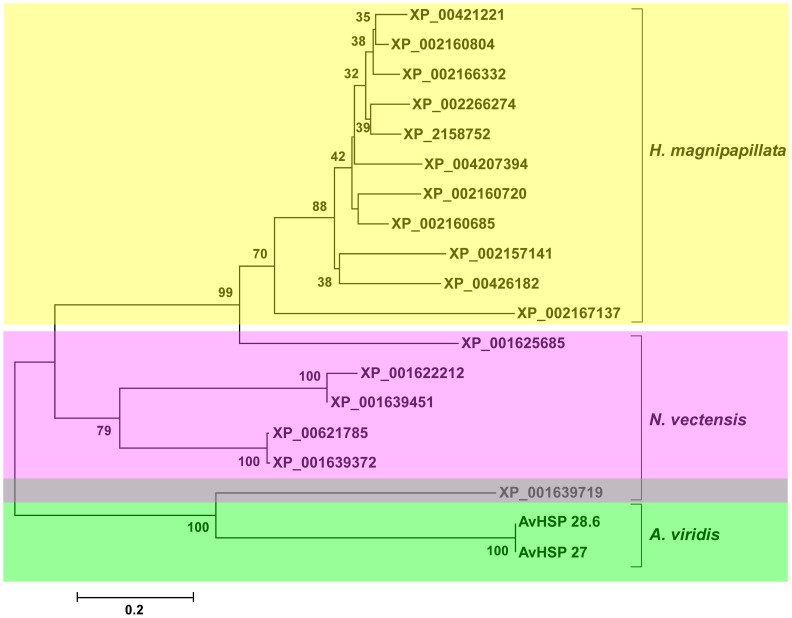
Neighbour-Joining (NJ) phylogenetic tree based on the ACD of *A. viridis*, *N. vectensis* and *H. magnipapillata*. The sequences used were obtained from GenBank at NCBI and listed in Table 2. The *p*-distance model was used to construct the phylogenetic tree. Internal branches were assessed using 1000 bootstrap replications. Bootstrap values less than 30% are not shown.

To investigate the evolutionary relationships among the cnidarians sHSPs, phylogenetic trees were constructed. The inferred NJ and MP trees showed a similar topology with branching according to taxonomic differences among *H. magnipapillata*, *N. vectensis* and *A. viridis*. Therefore, the phylogenetic analysis displayed the presence of three well-defined groups corresponding to species distinctions (Figure 5). Additionally, this topology highlights that the sHSPs from *A. viridis*, as a member of the Anthozoa class, are more primitive with respect to the more complex Hydrozoa class [52], [53].

As an exception to this rule, exclusively one sHSP from *N. vectensis* (XP_001639719) clustered with the sequences of *A. viridis*. This finding was surprising because *N. vectensis* belongs to the Anthozoa class. Notably, this sHSP is coded by the above described gene that shares a similar structural organisation with *A. viridis*.

From the topology of the tree, the intron dynamics of the *sHSP* family and the phylogenetic relationships among Cnidarians, it could be hypothesised that the *A. viridis sHSPs* likely represent the common ancestor from which *H. magnipapillata* genes directly evolved through segmental genome duplication.

Although both *A. viridis* and *N. vectensis* belong to Anthozoa, the fact that exclusively one *N. vectensis* sHSP both shares a similar gene architecture with *A. viridis* and also clusters in the protein alignment within Anthozoa raises a hypothesis for a common origin and phylogenic inferences. Evolutionary forces possibly acted to change the pattern of intron distribution within the Anthozoa class, and retroposition events may be responsible for the divergence of *N. vectensis* from *A. viridis*.

**Figure 6:**
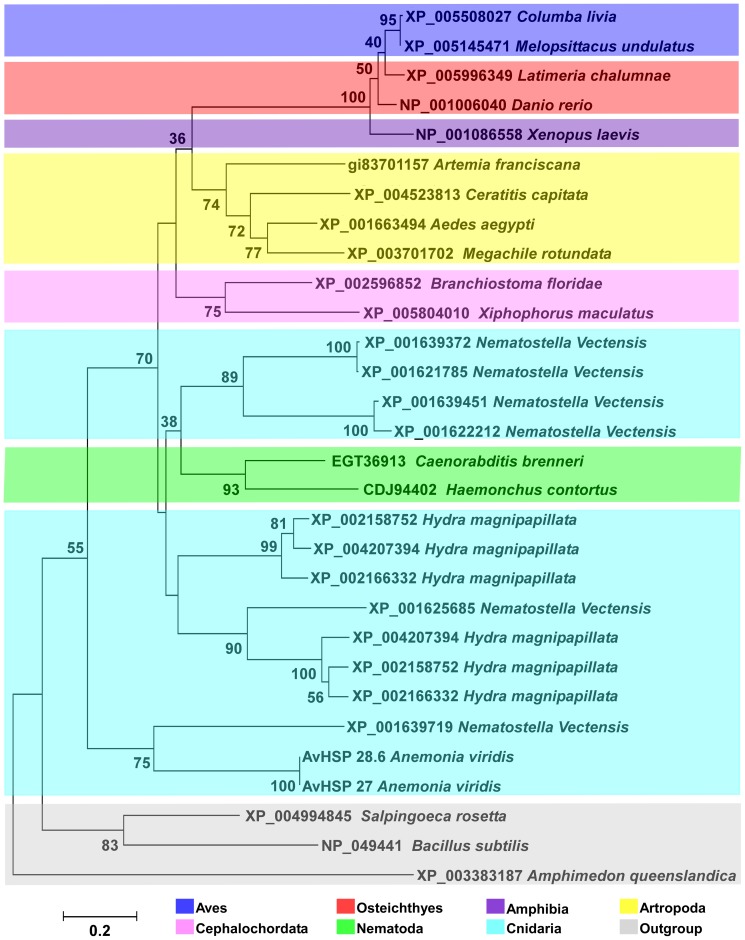
Neighbour-Joining (NJ) phylogenetic tree based on the ACD of cnidarians and other Eumetazoa. The tree was generated using MEGA 6.0 including sHSP from 19 different species ranging from aves to bacteria. All the sequences used were obtained from GenBank at NCBI and listed in Table 3. The sHSPs from *Salpingoeca rosetta* (Choanoflagellata), *Amphimedon queenslandica* (Porifera) and *Bacillus subtilis* (Bacteria) were used as outgroup. The *p*-distance model was used to construct the phylogenetic tree. Internal branches were assessed using 1000 bootstrap replications. Bootstrap values less than 30% are not shown.

The comparative analysis of sHSPs from other eumetazoans resulted in NJ and MP trees with similar topology, and the NJ tree is displayed in Figure 6. The reported taxonomic position of *A. viridis* and tree topology is similar to those constructed by Putnam et al. [29] with the ancestral status of the Anthozoa class basal to other Eumetazoans. Moreover, the other cnidarians sHSPs grouped with the main line comprising invertebrates and chordates. In this line, *H. magnipapillata* and *N. vectensis* are sister group of Nematodes; after this, the Arthropods and Cephalochordata branch off. Finally, the most modern sHSPs seem to be in Vertebrata, and the *N. vectensis* sHSP sequences show paraphyletic grouping in the tree, being clustered once with *Hydra* and once with Nematodes. Because Cnidarians are considered as a Metazoan ancestor and are known to show considerable molecular complexity [29], [54], [55], the evolutionary analysis with the sHSP family of other Eumetazoans provides additional data on cnidarians phylogenetic status.

### Tissue-Specific Gene Expression Pattern

To analyse the tissue distribution and expression profiles of *AvHSP28.6* and *AvHSP27* transcripts, total RNA was isolated from ectodermal- and endodermal-derived tissues of *A. viridis*. To monitor contamination of ectodermal or endodermal extracts, the transcripts for an isoform of carbonic anhydrase 2 (*CA2-M*) and hydroxymethylglutaryl-CoA reductase (*HMG-CoAR*) [36] were reverse transcribed and amplified in all samples as endoderm and ectoderm markers, respectively. *CA2-M* and *HMG-CoAR* amplicons were usually detected after 20 cycles in endodermal and ectodermal tissues, respectively, and limited cross-contamination was evident after 30 cycles of amplification (data not shown).

**Figure 7:**
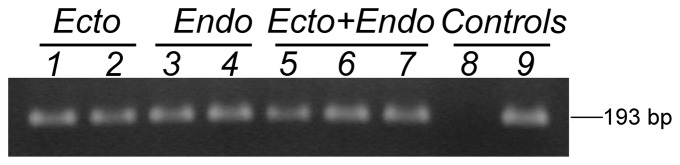
Expression patterns of *AvHSP28.6* in various tissues. Total RNA was extracted, reverse-transcribed and amplified from the endodermal (line 1 and 2) and ectodermal (line 3 and 4) cell layer of tentacles and body walls, while RNA isolated from pharynx (line 5), muscles (line 6) and gonads (line 7) was purified from both ectodermal and endodermal layers. Negative control (line 8) is also shown and total RNA from whole body specimens was used as a positive control (line 9).

The *AvHSP28.6* transcripts were detected in both ectodermal- and endodermal-tissues from the tentacles, body wall, muscle and pharynx (Figure 7), and it was also induced by different stressors as described below.

This result suggests that *AvHSP28.6* was constitutively expressed in adult sea anemones under normal physiological conditions.

By contrast, no *AvHSP27* transcripts were detected in the dissected tissues in both adult whole body cDNA libraries or in the stress conditions (data not shown). This result could indicate that this transcript is extremely underrepresented in our cDNA libraries, either because of low expression levels under physiological conditions or life-stage restricted expression. However, the alternative hypothesis is that this transcript is a pseudogene lacking regulatory elements controlling *AvHSP27* expression.

### Expression Profiles in Response to Thermal, Chemical, and Biotic Stresses

Previous studies have reported that the expression of sHSPs is rapidly up-regulated under conditions of stress to protect the cell from various types of damage [13], [49], and we noted that *AvHSP28.6* is a constitutive transcript present in all the analysed tissues.

Thus, to evaluate its involvement in the stress response, the the mRNA levels of *AvHSP28.6* were profiled after challenges with different abiotic/biotic stresses. The tentacles were selected as the target tissue because of the ability of the tissue to regenerate without impairment of viability after dissection.

**Figure 8:**
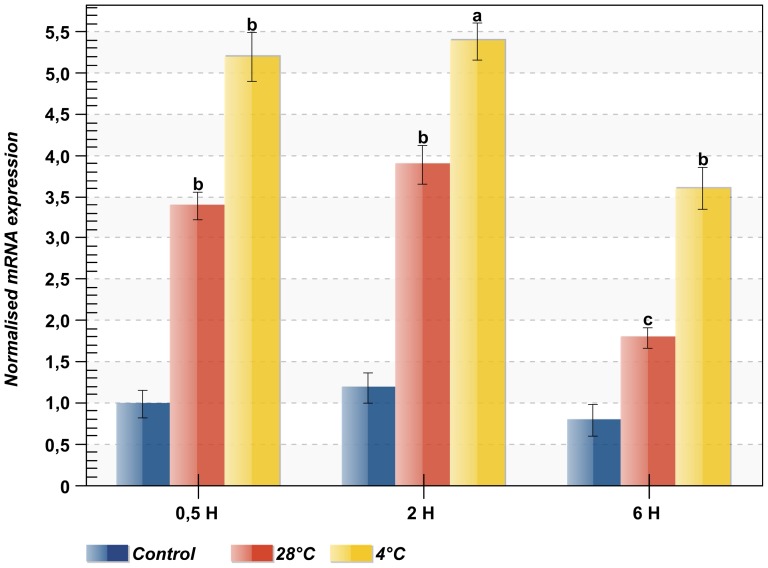
Expression profiles of *AvHSP28.6* in response to extreme temperature stress. Sea anemones were challenged by heat shock stress at 28°C and cold shock stress at 4°C. The tentacles were sampled at different time points post-challenge (0.5H, 2H and 6H). The expression level of *AvHSP28.6* was analyzed by qRT-PCR with 18S rRNA as reference. The results are represented as means ± S.D. (n = 3). Statistical analysis by one-way ANOVA with Tukey's post-test. Data with different letters are statistically different: a = *p*<0.0001, b = *p*<0.001, c<0.01.

When cells are exposed to thermal stress, many cellular proteins lose their structure and develop hydrophobic surfaces that tend to aggregate, leading to adverse cellular events [56], [57]. To cope with this stress, a large number of *HSP* genes were transcribed and expressed [58]. In our study, the exposure of sea anemones to two extreme temperatures (4°C and 28°C) resulted in a rapid induction of *AvHSP28.6* mRNA level (Figure 8). In response to heat shock challenge, *AvHSP28.6* expression was rapidly elevated to approximately 3.4-fold greater than the control level within 30 minutes. The *AvHSP28.6* transcripts accumulated to a maximum level at 2 h (3.8-fold) and then declined at 6 h. Even after 6 h, the expression was still higher than that of the control.

Compared to heat stress, few studies have reported the effects of cold stress on the expression of *sHSPs* [14], [59], [60]. The accumulation of *sHSPs* is considered part of the mechanisms responsible for tolerance to cold-stress injury in several organisms [61], [62]. The cold stress also induced a quick expression of *AvHSP28.6* in sea anemone. The transcript was over-expressed by up to 5.2-fold within 30 minutes.

The *AvHSP28.6* expression increased to a maximum level at 2 h (5.4-fold) and then reduced at 6 h. Even at 6 h, the expression remained higher than that of the control.

A few studies also reported examples of the down-regulation of the expression of *HSPs* in response to cold stress [63], [64]. Thus, it has been suggested that the patterns of *HSP* transcription varies substantially between organisms, tissues, contaminants, doses, periods of exposure and individual *HSP* genes [65]. Although the mechanisms for HSP activation in response to cold stress remain unaddressed, there are many similarities between the effects of heat and cold stress, including the denaturation of proteins, aggregation and cell death. Despite a few differences in fold change amplitude, a comparison of *AvHSP28.6* transcriptional activity in response to heat and cold shock reveals that the *AvHSP28.6* expression displayed similar profiles. We hypothesise that similar pathways were activated in *A. viridis* to protect and tolerate thermal stress by either heat or cold shock.

**Figure 9:**
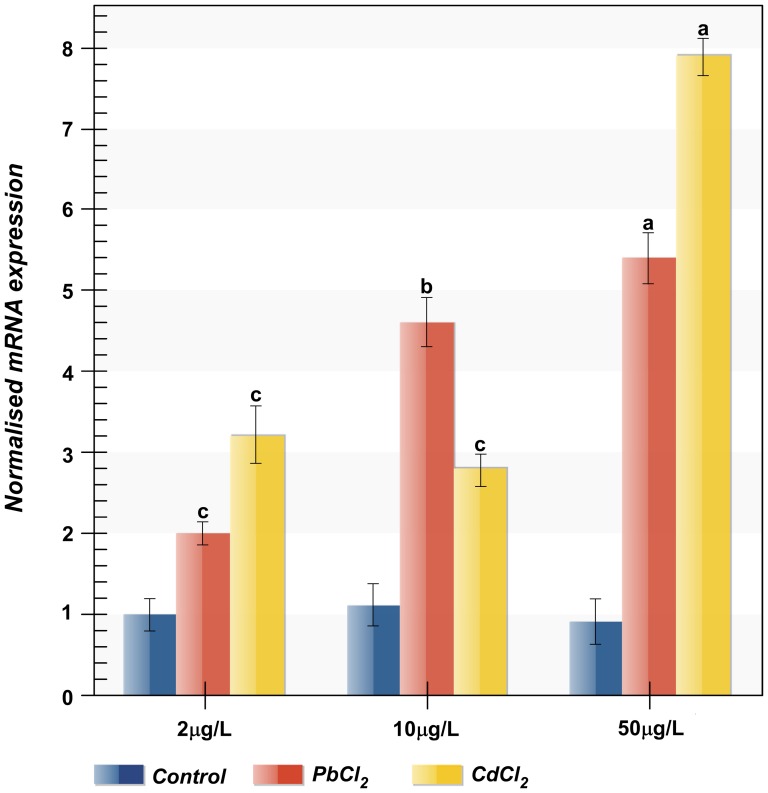
Expression profiles of *AvHSP28.6* in response to heavy metal exposure. Sea anemones were exposed to CdCl_2_ and PbCl_2_ at different concentrations for 18H. The expression level of *AvHSP28.6* was analyzed by qRT-PCR with 18S rRNA as reference. The results are represented as means ± S.D. (n = 3). Statistical analysis by one-way ANOVA with Tukey's post-test. Data with different letters are statistically different: a = *p*<0.0001, b = *p*<0.001, c<0.01.

Because heavy metal ions are known to impair cells viability, the effects of cadmium (Cd) and lead (Pb) exposure on *AvHSP28.6* gene expression were also analysed. As expected, the *AvHSP28.6* expression increased following the exposure to increasing concentration of pollutants(Figure 9). The transcript was up-regulated after exposure to 2, 10, and 50 µg/L PbCl_2_, and the highest expression (5.4-fold higher than that of the control group) was detected at 50 µg/L. Similarly, the *AvHSP28.6* expression was induced in response to CdCl_2_ exposure and was over-expressed by up to 8-fold at 50 µg/L.

Although the sHSP system is responsive to a wide spectrum of heavy metal pollutants, no study currently exists on the effects of heavy metals at a transcriptional level in sea anemones. However, our results are consistent with findings reported for the scallop *Argopecten irradians* [15], in which Cd and Pb also induced the expression of *HSP22*, and for the clam *Meretrix meretrix* [66], in which *HSP20* expression levels increased significantly after cadmium exposure. However, a recent study reported that in the clam *Sinonovacula constricta* [67], Cd down-regulates the expression of a *sHSP*, whereas lead maintained the role of a strong inducer. Even if the transcriptional patterns of *HSP*s in response to metals are variable, in our study, the *AvHSP28.6* gene was induced by both Pb and Cd at similar concentrations. Thus, such mRNA induction may functionally protect sea anemone from the heavy metal stress.

In aquatic environments, sea anemones and other animals belonging to different phyla are continuously exposed not merely to chemical and physical challenges but also to microbial pathogens. Cnidarians lack an acquired immune system, and the innate immunity is activated by pattern recognition receptors (PRRs), as Toll-like receptors (TLRs), that recognise a pathogen-associated molecular pattern (PAMP) including lipopolysaccharide (LPS)[68].

**Figure 10:**
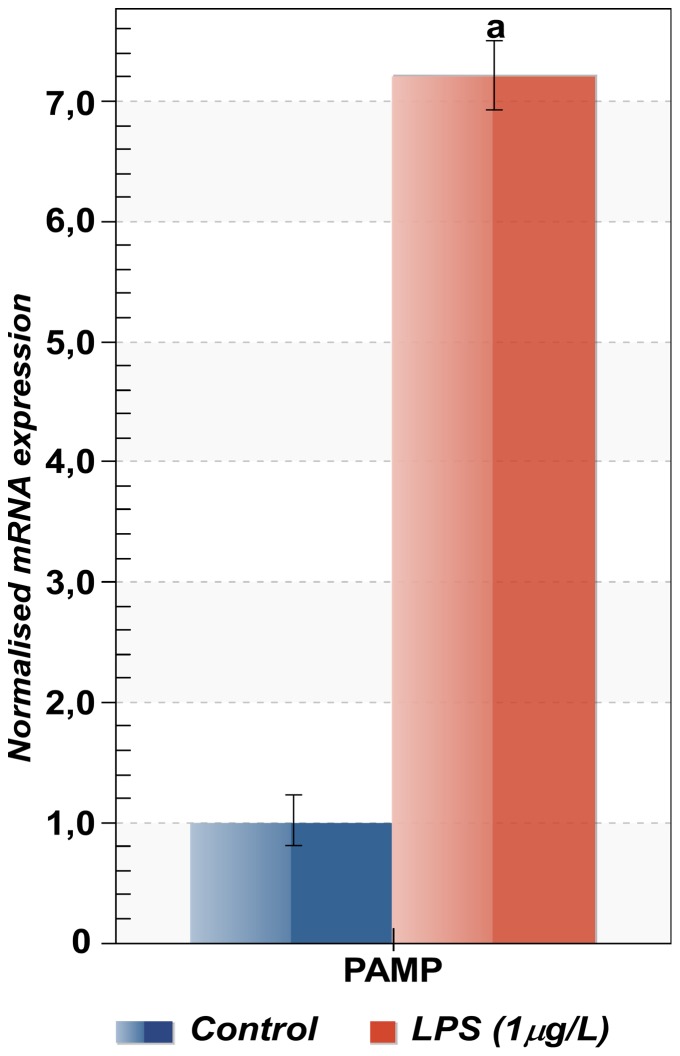
Expression profiles of *AvHSP28.6* in response immune stinulation. Sea anemones were exposed LPS (1 µg/ml) for 18H. The expression level of *AvHSP28.6* was analyzed by qRT-PCR with 18S rRNA as reference. The results are represented as means ± S.D. (n = 3). Statistical analysis by one-way ANOVA with Tukey's post-test: a = *p*<0.0001.

This prompted us to examine the amount of *AvHSP28.6* mRNA in sea anemones exposed to LPS which was used as an elicitor of the innate immune response. After exposure to LPS 1 µg/ml, *AvHSP28.6* mRNA expression resulted 7-fold higher than that of the control group (Figure 10).

Data focused on the involvement of the *sHSP* genes as a part of immunity response in non-model organisms are relatively limited and have emerged only recently. However, it has been shown that in aquatic animals, e.g. the bloody clam *Tegillarca granosa* [69], the disk abalone *Haliotis discus discus* [14] and the black tiger shrimp *Penaeus monodon* [70], the transcript levels of the *sHSP* genes were increased after the immune system was challenged.

In human the small HSPs are known to represent extracellular signals to cells of the innate system, as macrophages and dendritic cells, and to promote anti-inflammatory rather than pro-inflammatory responses [71].

Moreover, the p38MAPK signalling pathway plays a crucial role in the response to TLR activation and is required to coordinate the host response to pathogens [72], [73]. Although the direct involvement in the regulation of immune mechanisms in *A. viridis* is far from being fully described, the fact that sHSPs are part of p38MAPK pathways raises the possibility that the up-regulation of AvHSP28.6 expression could play a role in the defence mechanisms against infection.

## Conclusions

The *sHSP* genes from *A. viridis* clearly represent the ancestral form of Eumetazoan α-crystallin coding genes. Additionally, the similar intron/exon organisation of *H. magnipapillata* and *A. viridis sHSP* genes and the divergence of *N. vectensis*, enabled us to hypothesise that the *AvHSP28.6* representsthe common ancestor for the cnidarians *sHSPs* herein analysed. Moreover, it is likely that the presence of a conserved structure in the *sHSP* gene from *N. vectensis* is reminiscent of genes encoding alpha-crystallin in *A. viridis*.

Beyond the evolutionary perspectives, *AvHSP28.6* likely represents a component of the “defensome” [19], [74] in the genome of *A. viridis* since its mRNA was found to be overexpressed in response to environmental challenges, such as heavy metals, microbial products, and other exogenous factors, including global climate change. The data reported here may be used in both basic and applied research, and the sHSP of *A. viridis* may be employed as a potential biomarker indicating stress.

## Supporting Information

Figure S1 **qRT-PCR Amplification plot of** ***AvHSP28.6*** **and** ***AvHSP27*** **on genomic and cDNA templates.** No amplification curve for *AvHSP27* was obtained when cDNA was used as template.
(TIF)
