# Supplementary figures and images for: Characterization of Small HSPs from Anemonia viridis Reveals Insights into Molecular Evolution of Alpha Crystallin Genes among Cnidarians
Source: PLoS One. 2014 Sep 24;9(9):e105908. doi: 10.1371/journal.pone.0105908 (PMC4175457; doi:10.1371/journal.pone.0105908)

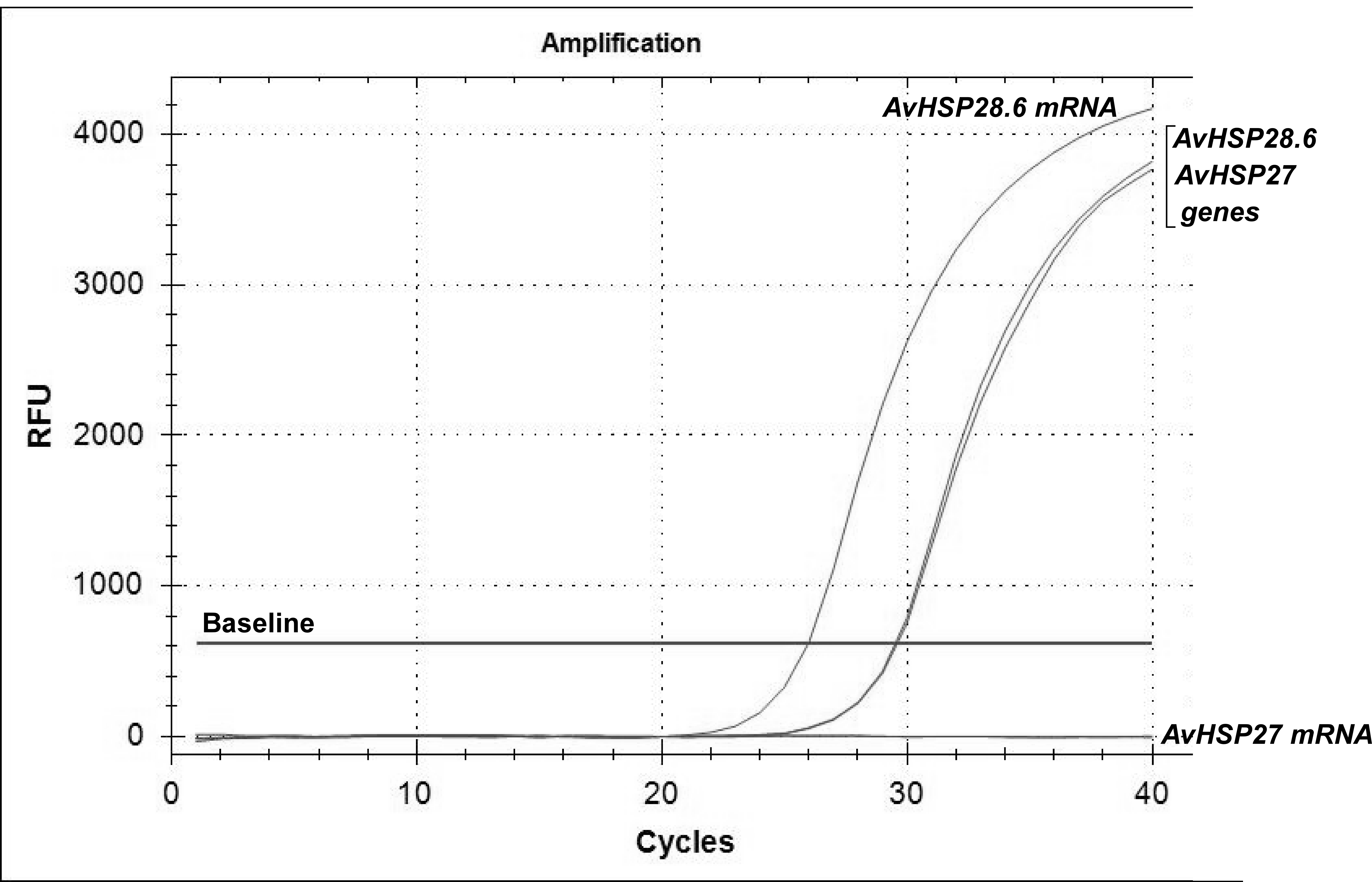

Supplement: Figure S1 — qRT-PCR Amplification plot of AvHSP28.6 and AvHSP27 on genomic and cDNA templates. No amplification curve for AvHSP27 was obtained when cDNA was used as template. (TIF) [file pone.0105908.s001.tif]
